# Multi-Hit White Matter Injury-Induced Cerebral Palsy Model Established by Perinatal Lipopolysaccharide Injection

**DOI:** 10.3389/fped.2022.867410

**Published:** 2022-06-06

**Authors:** Le Liu, Liwei Fang, Boyang Duan, Yue Wang, Zhenzhen Cui, Li Yang, De Wu

**Affiliations:** ^1^Department of Pediatrics, Pediatric Neurorehabilitation Center, The First Affiliated Hospital of Anhui Medical University, Hefei, China; ^2^Department of Pediatrics, Maternal and Child Health Hospital, The Affiliated Hospital of Anhui Medical University, Hefei, China; ^3^The Fourth Affiliated Hospital of Anhui Medical University, Hefei, China

**Keywords:** cerebral palsy, perinatal infection, white matter injury, animal model, electron microscopy

## Abstract

Cerebral palsy (CP) is a group of permanent, but not unchanging, disorders of movement and/or posture and motor function. Since the major brain injury associated with CP is white matter injury (WMI), especially, in preterm infants, we established a “multi-hit” rat model to mimic human WMI in symptomatology and at a histological level. In our WMI model, pups suffering from limb paresis, incoordination, and direction difficulties fit the performance of CP. Histologically, they present with fewer neural cells, inordinate fibers, and more inflammatory cell infiltration, compared to the control group. From the electron microscopy results, we spotted neuronal apoptosis, glial activation, and myelination delay. Besides, the abundant appearance of IBA1-labeled microglia also implied that microglia play a role during neuronal cell injury. After activation, microglia shift between the pro-inflammatory M1 type and the anti-inflammatory M2 type. The results showed that LPS/infection stimulated IBA1 + (marked activated microglia) expression, downregulated CD11c + (marked M1 phenotype), and upregulated Arg 1 + (marked M2 phenotype) protein expression. It indicated an M1 to M2 transition after multiple infections. In summary, we established a “multi-hit” WMI-induced CP rat model and demonstrated that the microglial activation correlates tightly with CP formation, which may become a potential target for future studies.

## Introduction

Cerebral palsy (CP) is a permanent (not unchanging) motor dysfunction and postural abnormality caused by non-progressive disturbance or developing abnormality of the immature brain, with an average prevalence of 1.5–3.0‰ in neonates (1). In a meta-analysis of China, the prevalence of CP over the 32 years from 1988 to 2022 was 2.07‰ among 0–18 years old (2). Motor dysfunction, which is the main syndrome of CP, is often accompanied by intellectual disability, behavioral problems, sensation or perceptual disorders, epilepsy, etc. (3, 4). For now, there is no consensus on the upper age limit of brain disturbances for the definition of CP. Children over 2 years of age who acquired brain damage may already have certain skills, and the resulting disability can be very different (5). Due to the high plasticity of the developing brain, therapies and rehabilitation of infants with CP should be started as soon as possible (6). For children with severe cerebral palsy, costly and long-term treatments are too burdensome for the whole family to carry on. Identifying the etiology can help diagnose CP as early as possible and even prevent it from occurring.

As a clinical syndrome, CP etiology varies, but the precise cause remains obscure. The risk factors affecting the central nervous system (CNS) in early development are mainly divided into the following categories: external environment, the physical condition of the mother, prenatal, perinatal, as well as postnatal risk factors (1). Premature birth, hypoxia–ischemia, placental insufficiency, and perinatal infection, such as chorioamnionitis, are well-studied causes of CP (7). Among the various CNS damages caused by risk factors mentioned above, white matter injury (WMI) is the major one that leads to CP. Especially in very premature infants born before 28 weeks, the incidence of CP is as high as 13–17% (5, 8). WMI is characterized by hypomyelination, astrogliosis, and microgliosis. Due to the vulnerability of myelin sheath, many risks including cerebral ischemia/reperfusion and perinatal infection could contribute to the pathogenesis of WMI, making it hard to maintain neural signaling stability (7, 9, 10). So, commonly used rat models of WMI mainly include perinatal hypoxia and inflammation. Lipopolysaccharide (LPS), the main component of the gram-negative bacterial cell wall, is widely used in the induction of inflammation, although inflammation is often induced prenatally, applied alone (11, 12) or in combination with hypoxia (13–15). Few studies of CP have applied LPS both prenatally and postnatally.

Microglia are immunocompetent cells that reside in the CNS representing 10% of brain cells (16). They have neuroprotective and neurotoxic effects and are considered “CNS macrophages” (17). They can be activated into “classical activation” M1 type and “surrogate activation” M2 type in different circumstances. M1 polarization results in the release of inflammatory factors such as IL-6, IL-1β, and TNF-α, triggering an inflammatory cascade and aggravating brain damage, while M2 microglia secrete anti-inflammatory cytokines and neurotrophic factors to reduce the inflammatory response, protect neurons, and promote tissue repair (18, 19). When infection occurs, increased IL-6 and TNF-α induce microglial activation to the M1 type and release more pro-inflammatory factors, amplifying the inflammatory cascade by acting on nearby non-activated microglia, causing a vicious cycle that contributes to secondary brain injury (20, 21). Studies found that activated microglia impede oligodendrocyte lineage progression, reduce the production of myelin binding protein, and impair myelination (22–24). One of the theories showed that microglial activation would enhance the notch signaling pathway and inhibit oligodendrocyte differentiation and myelination (25–27). However, there are still many unknowns worth exploring.

As far as we know, microglia function as a double-edged sword. The role of microglial activation and polarization in the formation of cerebral palsy needs to be further studied. Here, we established a “multi-hit” WMI-induced CP rat model to investigate the role of microglia activation and imbalance in the formation of CP, providing a potential target for the treatment of CP.

## Materials and Methods

### Animals and Models

**FIGURE 1:**
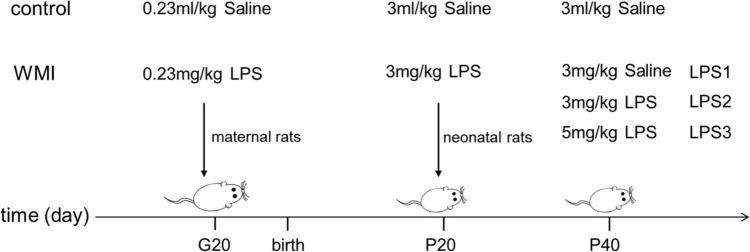
Administration methods for animal models. The image shows the time and dose of intraperitoneal injection of drugs in each group. Pregnant rats were given the first injection at 20 days of gestation (G20), and the second injection of LPS was conducted on neonatal rats at postnatal day 20 (P20) to construct the WMI animal model. We divided experimental mice into three groups and again intraperitoneally injected LPS consisting of low to high concentration gradients at postnatal 40 (P40), to mimic the adverse effects of multiple infections early in development.

Healthy Sprague–Dawley rats of childbearing age were purchased from Beijing Vital River Animal Center, SCXK: (Jing) 2016-0006, including 10 males of 290–300 g and 20 females of 240–250 g. The experimental animals were housed in a standard animal center of (23 ± 2)°C and 55% humidity, with free access to food and water, maintained on a diurnal rhythm of 12 h light and 12 h dark. After 1 week of adaptive feeding, rats were co-caged according to the ratio of male and female 1:2, lights off for 12 h, and the vaginal smears were examined the following day to count as gestational day 1 when sperm plugs were found. Fourteen pregnant rats were randomly divided into two groups, one group was intraperitoneally injected with 230 μg/kg LPS (Sigma, 16511, Serotype typhimurium, United States) suspended in saline at 20 days of gestation (G20) and other group was injected with the same volume of saline. The LPS dose (230 μg/kg) was chosen based on preliminary studies. Then, the respective offspring were treated as experimental and control groups (the mortality of the control group was 2.3%, while the experimental group was 24.6%). The pups in the experimental group received an intraperitoneal injection of 3 mg/kg LPS in saline at a postnatal age of 20 days (P20), while the control group received 3 ml/kg saline. To mimic the effects of repeated inflammatory infection in the early stage of development, we divided the experimental group into three groups at 40 days of age (P40), and the low-dose group was injected with saline, the middle dose group was injected with 3 mg/kg LPS, the high-dose group was injected with 5 mg/kg LPS, and the control group was injected with an equal amount of saline (Figure 1). The experiments were approved by the Experimental Animal Ethics Committee of Anhui Medical University: LLSC 20190126.

### Neurobehavioral Tests

#### Berderson Score

It is also known as the tail suspension test, and the postural reflex is observed by grasping a rat’s tail at a certain distance from the ground.

1. Hold the rat by the tail and keep it 0.5 m high above the table. Record the scores as follows: 0 = both limbs symmetrically extended forward; 1 = internal rotation of shoulder and adduction of limbs.
2. Place the animal on a rough surface. Record the scores as follows: 0 = a strong grasp on the rough surface with good resistance when pushed; 1 = a slight resistance was only seen in one paw; 2 = no resistance when pushed in one direction.
3. Place the rat’s forelimbs on a metal net and lift the rat gently to check the muscle strength of the forelimbs. Record the scores as follows: 0 = both forelimbs grasp the net firmly; 1 = only one limb grasps the net; 2 = both limbs cannot hold the net.

Use the sum of the assessment scores from each task as the final assessment score (28). The higher the score, the more severe the rat’s behavior disorder.

### Balance Beam Test

The balance beam is a test of motor coordination and balance in rodents. It evaluates sensorimotor integration by examining limb function.

1. Ensure that the apparatus consists of a 2.5 cm wide and 80 cm long beam and is 10 cm above the floor.
2. Place a white noise generator and bright light source at the start of the beam. The noise and light were used to motivate the rat to traverse the beam and reach the terminus.
3. Record the scores for each performance as follows: 0 = balances with steady posture; 1 = grasps the side of the beam; 2 = hugs beam and 1 limb fall off beam; 3 = hugs beam and two limbs fall off beam; 4 = attempts to balance on beam but falls off; and 5 = falls off, no attempt to balance. The score is proportional to the severity of balance impairment (29).

### Western Blot

Forty-eight hours after the third administration, we anesthetized the pups by intraperitoneal injection of 10% chloral hydrate and quickly removed brain tissues (kept in a –80°C freezer). We weighed an appropriate amount of brain tissue, thoroughly ground it by adding protein lysis solution (10 UL PMSF + 1 ml RIPA), and centrifuged the brain homogenate at 12,000 r/min for 10–15 min. The supernatant was aspirated and centrifuged again for 10 min, SDS-PAGE sample loading buffer (5×) was proportionally added, boiled in a metal bath at 99°C for 5–10 min for protein denaturation, and stored in a freezer at –80°C after recovery to room temperature. Then samples were sequentially added into wells of a 10–12.5% SDS-PAGE gel. After complete separation of the maker, it was blocked for 1–2 h at room temperature in TBST, following a constant current wet rotation at 200 mA and overnight incubation with rabbit anti-β-actin(1:5000), IBA1(1:2000), CD11c(1:1500), and Arg1(1:2000) at 4°C (see Table 1 for detailed parameters). Membranes were washed three times for 10 min each in TBST the next day, incubated with secondary antibody for 1 h at room temperature, and again washed three times for 10 min each in TBST. The grayscale values of each band were exposed and developed on a developing machine. Results were analyzed by Image J.

**TABLE 1:** List of antibodies used in this study.

Primary/secondary antibody	Species	Sources	Catalog number	Usage	Dilution factor
IBA1	Rabbit	Abcam, United States	ab178846	WB/IHC	1:2000
Arg1	Rabbit	GenTex, United States	GTX109242	WB	1:2000
CD11c	Rabbit	Affinit, China	7853	WB	1:1500
β-actin	Rabbit	Zenbio, China	380624	WB	1:5000

### Hematoxylin–Eosin Staining

The brain tissues were fixed in 4% paraformaldehyde for 24 h, processed, embedded in paraffin, and subsequently cut into 4–8 μm-thick sections. Following deparaffinization, tissue samples were hydrated, hematoxylin stained, differentiated with bluing reagent, eosin stained, and dehydrated. Sections were air-dried, coverslipped, and photographed under a light microscope (Motic AE2000, Japan). Typical sections of the hippocampus and cerebrum were made in each group of animals.

### Immunohistochemistry

The brain tissues were fixed in 4% paraformaldehyde, washed, dehydrated, transparent, waxed, embedded, and sliced. Then, they were gradient dewaxed, antigen repaired, cooled to room temperature, and washed in a wet box with PBS 3 times for 5 min each. Each section was dripped with endogenous peroxidase and incubated for 20–30 min in a 37°C incubator. It was washed with PBS 3 times, 5 min each. Next, we added primary antibody dilutions to each section and placed them in a humidified box at 4°C overnight. After returning to room temperature for 30 min, it was washed with PBS three times for 5 min each. The second antibody was added and incubated at 37°C for 30 min. Chromogenic time (3–5 min) was determined according to the titer intensity of the antibody. The newly prepared DAB chromogenic solution was added dropwise until we observed the specific staining under a microscope. The reaction was stopped with distilled water. After counterstaining with hematoxylin for 1 min, rinsed with running water, dried naturally at room temperature, the sheets were sealed with neutral gum, and baked overnight in an oven. Images were taken under a light microscope (Motic AE2000, Japan). Five regions of each specimen were randomly collected to calculate the average number and the average optical density of positive cells by Image J.

### Electron Microscopy

Three rats from each group were anesthetized intraperitoneally with 10% chloral hydrate, and the heart was fully exposed by thorax opening. We quickly removed the brain after fully injecting a mixture of paraformaldehyde and glutaraldehyde into the heart. Then the tissue of the frontal cortex was cut into 1 × 1 × 1 mm^3^ blocks on ice and fixed in a 2.5% paraformaldehyde glutaraldehyde solution. After rinsing, fixing, dehydrating, embedding, and sectioning into 70 nm, uranyl acetate and lead tartrate were double-stained and photographed under a transmission electron microscope (Thermoscientific Talos L120C G2, United States).

### Statistical Analyses

Statistical analysis of mean differences between groups was performed by using one-way ANOVA (followed by Bonferroni test) or unpaired Student’s *t*-test. Non-normal distribution data were analyzed by Mann–Whitney *U* test. We used GraphPad Prism (v.9.1.0.221) for all the statistical analyses. *P* < 0.05 was considered significant and all *p*-values and *n* values are indicated in figure legends.

## Results

### Behavior Tests on Experimental Rats

**FIGURE 2:**
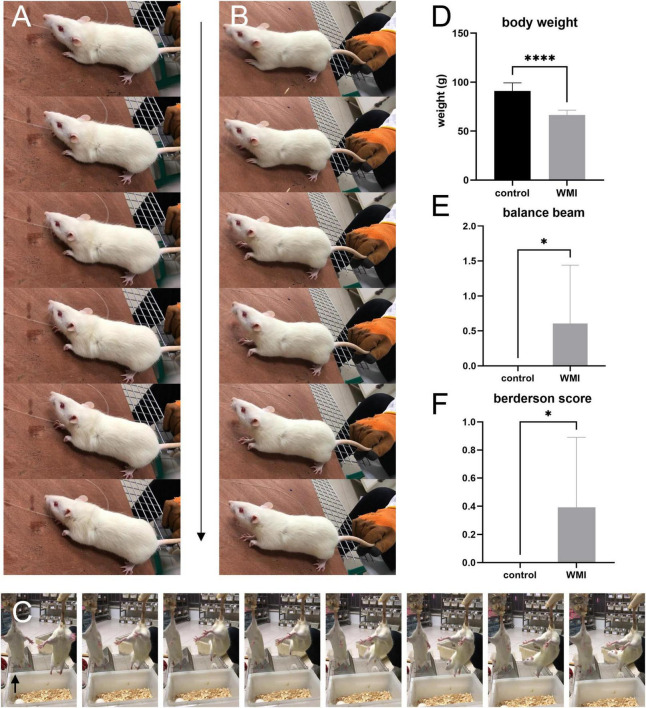
The images and graphs of behavior experiments (*n* = 12 rats for the control group, *n* = 28 rats for the WMI group). Frame-by-frame photos were taken at 5 frames per second. **(A,B)** A typical rat of the WMI group. **(C)** WMI group on the left and control group on the right. The range of motion in the WMI group was relatively smaller than the control one. **(D)** The bodyweight of the WMI group was lower than that of the control group (*p* < 0.0001 by unpaired two-tailed *t*-test). Data are presented as the mean ± SE. **(E)** In the balance beam experiment, the WMI group scored higher than the control group (*p* = 0.0121 by Mann–Whitney *U* test). **(F)** Berderson score from the rats in the control and WMI groups. The comprehensive score of the WMI group was higher than that of the control group (*p* = 0.0170 by Mann–Whitney *U* test). *P < 0.05, ****P < 0.0001.

After 3 days of intraperitoneal injection of neonatal rats, we weighed the rats and ran behavior tests on both WMI and control groups (*n* = 12 rats for the control group, *n* = 28 rats for the WMI group). The average bodyweight of the control group was 91.98 ± 2.41 g, and the WMI group was 66.51 ± 0.91 g, the difference indicating that the rats in the WMI group grow slower than normal ones (Figure 2D). In the balance beam test, WMI rats showed weaker body control and exhibited more retardation on how to balance but just hug the beam, and so scored higher than the control group (*P* = 0.0121 < 0.005; Figure 2E). In the Berderson score, some rats in the WMI group showed spasms/paresis of the upper limb and limited movement. When we placed them on the rough table, a slight resistance was only seen in one paw. The rat had mild paresis of the left upper limb, whereas the right forepaw and both hind paws grasped forcefully. The left forepaw spasticity was evident on movement (Figures 2A–B). We held the rats by the tail and kept them 0.5 m high above the ground. It was noticed that the rat in the control group symmetrically extended both its limbs forward, but the rat in the WMI group showed a slight internal rotation of the left shoulder and adduction of the left upper limb (arrow in Figure 2C). It also moved rigidly and showed an incoordinate postural reflex, while the rat in the control group showed normal spontaneous movement (Figure 2C). Taking these tests together, the total Berderson score was higher than that of the control group (*P* = 0.0170 < 0.05; Figure 2F).

### Histopathological Results of the Brain Tissue

**FIGURE 3:**
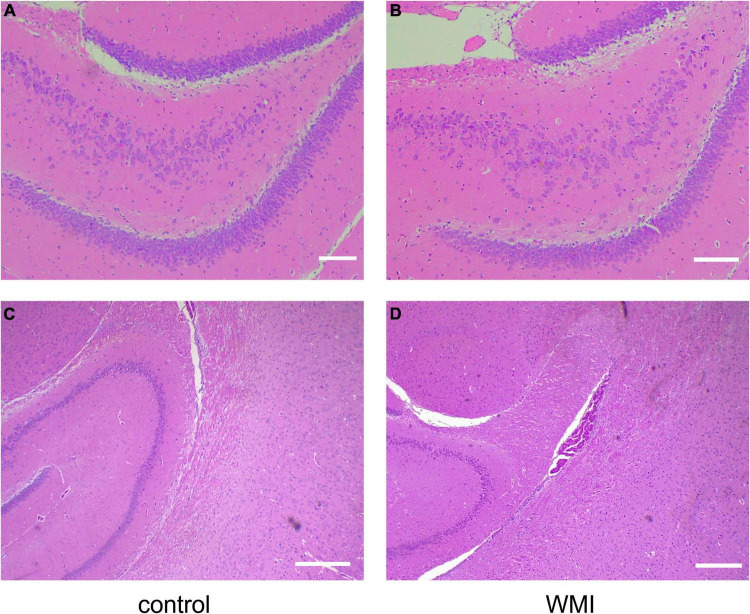
Hematoxylin-eosin staining of rat hippocampus (*n* = 2–3 brains per group). **(A)** The hippocampus of normal rats in the control group showed many neurons with a close and orderly arrangement. Scale bar = 100 μm. **(B)** In the WMI group, nerve cells in the hippocampus were swollen, reduced in number, and disordered. Scale bar = 100 μm. Compared with the control group **(C)**, hippocampal neurons in the WMI group **(D)** were sparse and the peripheral nerve fibers were disordered. Scale bar = 200 μm.

**FIGURE 4:**
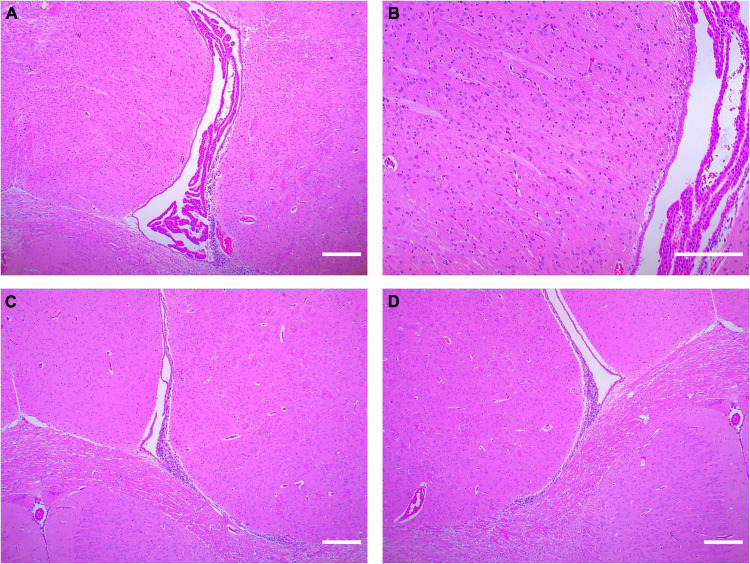
Hematoxylin-eosin staining around the lateral ventricle in rats (*n* = 2–3 brains per group). **(A)** The rats in the control group (P20) had normal lateral ventricles, developed choroid plexus, and abundant and orderly nerve fibers in the white matter adjacent to the lateral ventricles. **(B)** Periventricular white matter at 4× magnification. **(C,D)** In the WMI group (P20), lateral ventricle development was slower, choroid plexus atrophy and nerve fibers were sparse. Scale bar = 200 μm.

We took the brain tissue of rats for hematoxylin-eosin staining and observed the changes in cerebral structure in the experimental group from the histopathologic level. Compared with the control group, the WMI group had a fewer and looser arrangement of neurons, unclear cell boundaries, irregular arrangement of peripheral white matter nerve fibers, and massive infiltration of inflammatory cells (Figure 3). In normal rats of the control group, the choroid plexus was well developed in the lateral ventricle, and periventricular nerve fibers were orderly arranged, while in the WMI group, the white matter of the same part was looser, and nerve fibers were more scattered and less in number (Figure 4).

### Scanning Electron Microscopy Observations

**FIGURE 5:**
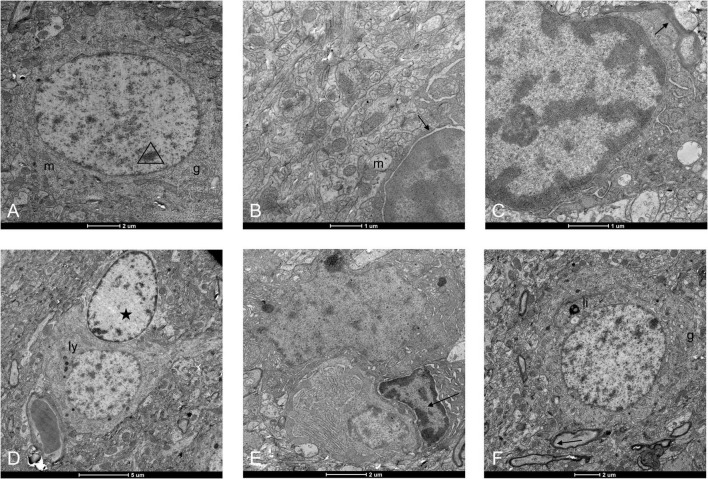
Ultrastructure of neural cells under an electron microscope in the WMI group. **(A)** Neuronal cell injury initiates programed cell apoptosis, and chromatin in the nucleus begins to condense. Organelles such as mitochondria (“m”)and Golgi apparatus (“g”) are normal. Scale bar = 2 μm. **(B)** Nucleolus pyknosis, nuclear membrane, and nucleolar space enlarges (arrow), and mitochondrial swelling. Scale bar = 1 μm. **(C)** The oligodendrocyte has a darker cytoplasm than the surroundings and is in attachment to a myelin sheath (arrow). Its nucleus was deformed, and chromatin was highly condensed. Mitochondria appeared vacuolated with excessive swelling, even some floccule appeared in the lumens. Scale bar = 1 μm. **(D)** A neuron in contact with the blood vessel was degenerative with more intracytoplasmic lysosomes (“ly”). An astrocyte came to repair it (pentacle). Scale bar = 5 μm. **(E)** The microglia (arrow) phagocytized a neural cell and a plasma cell. The specific cell morphology of the nerve cell above disappeared, but the plasma cell below was still visible with relatively intact cell morphology, and there was also abundant Golgi in the cytoplasm. Scale bar = 2 μm. **(F)** Lipofuscin (“li”) appeared within the cytoplasm of an injured neuron and there was some stunted myelin in the periphery. Myelin damage (arrow). Scale bar = 2 μm.

Deep into the cellular level, the neural cells of the WMI group were damaged. Cells started showing chromatin condensation with little changes in the organelles (Figure 5A). The karyotheca disintegrated, chromatin highly condensed, mitochondria were swollen, and even vacuolated degeneration appeared (Figures 5B,C). We captured an astrocyte surrounding the degenerating neuron and tried to repair it (Figure 5D). In the late phase of tissue injury, some microglia exhibit active phagocytic capacity, processing the necrotic neural cells (Figure 5E). After microglial activation, jugged1 expression is upregulated and its interaction with notch1 receptors on oligodendrocyte precursor cells is enhanced, which in turn inhibits oligodendrocyte maturation and myelination, hampering the normal conduction of nerve fibers (26). We could see a degenerating neuron with mildly condensed chromatin and partly fragmented mitochondria. The surrounding myelin sheath was slightly damaged (arrow), and the thickness of the peripheral myelin sheath was thinner than normal (Figure 5F).

### Abnormal Activation of Microglia

**FIGURE 6:**
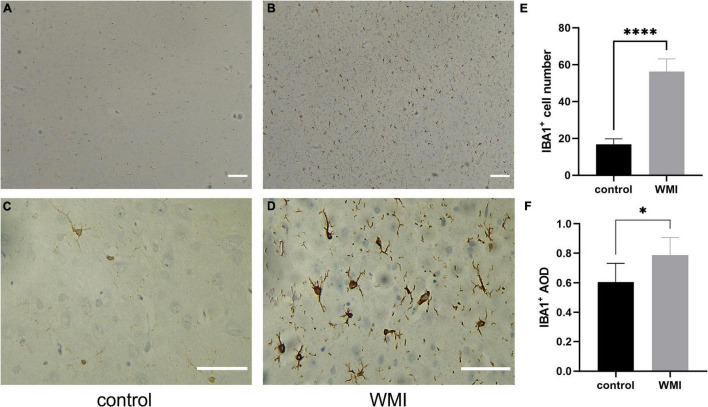
Microglial activation in WMI group. **(A,B)** The control group is on the left and the WMI group is on the right. Scale bar: 200 μm. **(C,D)** A magnified version of A and B. IBA1 + microglia were morphologically diverse, with most cells extending processes. Scale bar: 100 μm. **(E,F)** Quantitative analysis of the IBA1-positive areas in each group (2–3 sections were evaluated per pup). Five regions of each specimen were randomly collected to calculate the average number of positive cells and the average optical density. Data are presented as the mean ± SE. Compared to the control group by *t*-test, *P < 0.05, ****P < 0.0001.

Microglia, as the resident immune cells in the brain, we speculate that it will be activated during cerebral white matter injury, and different degrees of infection will cause different degrees of cell activation. We selected a specific protein, ionized calcium-binding adaptor molecule1 (IBA1) (30), to trace the microglia. In the WMI group, microglia were numerous and morphologically diverse, with most cells extending processes. However, in the control group, microglia appeared less active (Figures 6A–D). The average number of IBA1 + cells was 56.2 ± 3.1 in the WMI group and 16.8 ± 1.3 in the control group (*P* < 0.0001), which illustrates a greater number of activated microglia in the experimental group (Figure 6E). The expression of IBA1 was also higher as compared to that of the control group (Figure 6F).

**FIGURE 7:**
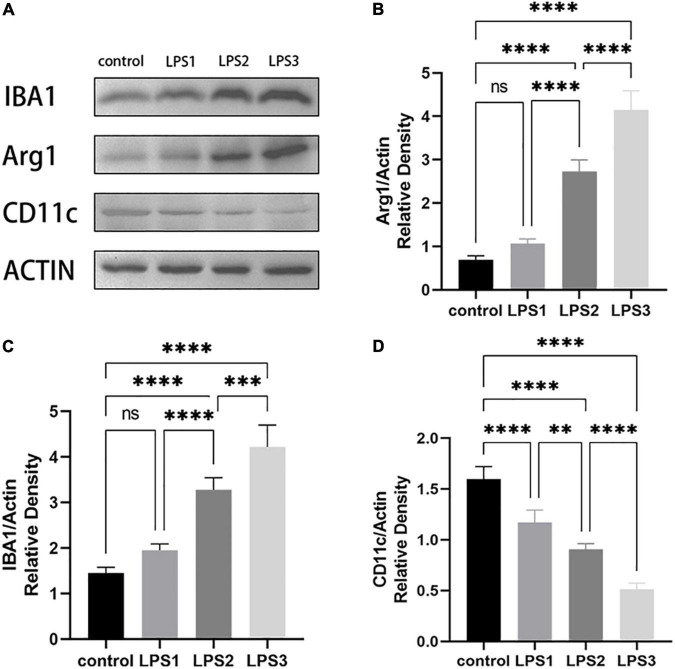
Western Blot of Arg1, CD11c, and IBA1 (*n* = 2–3 brains per group). **(A)** WB bands of the control group, low dose, medium dose, and high dose WMI + LPS groups. **(B–D)** The histograms represent the normalized relative density of Arg1(B), CD11c(C), and IBA1(D) between the control group and experimental groups. Data are presented as the mean ± SE. Compared the mean of each group with the mean of every other group by the Bonferroni test. ns > 0.05, **P < 0.01, ***P < 0.001, ****P < 0.0001.

Then, we divided WMI mice into three groups and again intraperitoneally injected LPS to simulate the effects of multiple infections after birth on WMI-induced cerebral palsy (Figure 7A). One-way ANOVA was used to compare the three experimental groups. The differences between the groups were significant in Arg1(*F* = 177.6, df = 3, *P* < 0.0001), IBA1(*F* = 94.78, df = 3, *P* < 0.0001), and CD11c(*F* = 114.7, df = 3, *P* < 0.0001). Then, the mean of every group was compared by the Bonferroni test. The expression levels of Arg1 and IBA1 in the LPS2 and LPS3 groups were higher than those in the control group, while both LPS1 groups showed no significant differences when compared with the control group (Arg1: *P* = 0.2581 > 0.05; IBA1: *P* = 0.0861 > 0.05). But there were significant differences among different dose groups. The expression levels of Arg1 and IBA1 in the LPS3 group were higher than LPS2 group, and in the LPS2 group, it was higher than in the LPS1 group. It showed that the expression level was increased with the elevating concentration of LPS, suggesting a certain dose-dependent effect (Figures 7B,C). The expression level of CD11c in LPS groups was lower than that in the control group. With the increase in LPS dosage, the expression of CD11c decreased gradually (Figure 7D). In different microenvironments, microglia can be activated into M1 and M2 types, respectively, and normally, the conversion between the two maintains a balance. The expression of CD11c, an M1-specific protein, gradually decreased with an increase in LPS concentration, while Arg1, an M2-specific protein, showed a gradual increase. It was seen that when WMI rats were infected early in development, microglia were not only activated but also polarized toward M2 type cells, more toward anti-inflammation to protect cerebral white matter.

Combined with the above microscopic examination and behavior experimental observation, the experimental group showed behavioral abnormalities, delayed neural development, and periventricular injury histologically. In the present results, we successfully established an animal model of WMI-induced cerebral palsy. Substantial microglial activation and a trend of microglia protecting the neural tissue after multiple injections of LPS were also observed. However, the specific roles of microglia require further studies to explore.

## Discussion

There are many patients with CP who did not manifest significant limb paralysis symptoms in the primary injury stage but gradually appeared in the subsequent development stage. For example, dyskinetic CP caused by bilirubin encephalopathy, usually appears as paralysis clinical symptoms 6 months after birth; mild CP caused by premature birth and intrauterine hypoxia need to be diagnosed at over 2 years old, some even reach 4 years old (31); and not all the WMI patients will finally develop CP based on clinical practice (32). Brain injury from a single etiology does not seem to be enough to cause CP, and the post-injury effects may play an important role. In animal studies, LPS-preconditioning on neonates may prime the brain to be more sensitive to subsequent inflammatory hits (33, 34). An observational study in humans also supports this possibility (35). An acute (non-progressive) brain interference in development is one of the causes that lead to brain damage, but two or more interferences are associated with a higher risk of damaging the brain in preterm newborns (36–38). It suggests that the onset of cerebral palsy is the result of “multi-hit” (39), including cerebral injury and biological or environmental adverse factors (such as inflammatory infections early in development) during the perinatal period. In the present study, we injected maternal rats with LPS intraperitoneally in the early stage of pregnancy and again administered two LPS in neonatal rats at P20 and P40 to create a WMI-induced cerebral palsy animal model, referring to the experimental methods of Felipe Stigger and Yanrong Hu et al., with some modifications (13, 14). Under some special circumstances, an inflammation-induced environment protects the brain from a secondary hit (40). Researchers applied a harmless dosage of LPS 24 h before a large dosage of LPS administration to make it resistant to brain injury (41). Repeated LPS administration would attenuate when the time interval is 24–72h, and less than 6 h or more than 72h prior to a second LPS exposure would exacerbate brain injury, even though the dose is not enough to cause injury alone (34, 41). Based on the “multi-hit” theory, we applied LPS injection both prenatally and postnatally to get a brain injury model more easily and more clinically. These models manifested paresis of limbs/tail, incoordination, and reduced activity, which mimic the symptoms of CP. It indicates that WMI would trigger CP under multiple effects, such as perinatal infection.

We hypothesized that microglia activation and polarization play an important role in the pathogenesis of WMI-induced CP. Known as CNS macrophages, microglia show similar bio functions but different cellular origins with the macrophage. When infection occurs, the activated microglia transform toward an amoeboid morphology, phagocytes the broken neural cells, and release cytokines, or oxidases to mediate the apoptosis of pre-oligodendrocytes (19, 42, 43). Microglial activation can also inhibit the differentiation and myelination of oligodendrocytes by enhancing the notch signal-pathway (25–27). Microglia clusters near periventricular axonal crossroads of premature infants, help prune the axonal bundles (44–46); some even colonize the subplate and synchronize with the synaptogenesis and growth of subplate neurons (7, 47). These signs reveal that microglia not only regulate the microenvironment of CNS but also participate in the formation of neural fibers in the early developmental stage. Through immunohistochemistry and western blotting, we screened tremendous activation of microglia in the experimental group, which is in line with the existing research points.

As mentioned before, activated microglia can be classified as pro-inflammatory M1 type and anti-inflammatory M2 type. Some researchers called “resting microglia” as M0 type or attenuated M2 type, but the debate remains (18). Based on the high plasticity and rapid transition between different phenotypes, others propose calling it “never-resting microglia” may be better (48). Anyway, such an M1/M2 classification helps researchers judge microglial states and microenvironment around more conveniently. In our experiments, the protein marked M1 got a lower expression in the WMI + LPS group while M2 specific protein showed an increased expression. The results showed an M2 polarization in P40 rats. D.M. Norden et al. demonstrated that repeated LPS injections (at regular intervals) did not magnify the inflammatory reaction, but instead stimulated the neuroprotective effect of microglia, suggesting that the protective effect of microglia may be time-dependent (49). A brain injury could surely activate microglia, and the M2-to-M1 microglial transition only occurred in the acute phase, but after a certain time, M2 started to dominate (27, 50). We speculate that in our WMI-induced cerebral palsy model, such a time demarcation also exists. Before the time point, risk factors such as infection can aggravate white matter damage and microglia mainly exert neurotoxic effects. After the time point, the microglia mainly exerts anti-inflammatory effects; however, brain damage at this moment is already irreversible. So, finding this time demarcation would play a key role in the future treatment of brain injury, which needs further studies.

In recent years, many researchers were keenly aware of the link between gender and cerebral inflammation. Both in animals and humans, it has been found that males are more likely to show increased expression of inflammatory indicators and corresponding neurobehavioral symptoms in inflammatory encephalopathy, while females show resilience to neuroinflammation and less severe phenotype (51–53). Lin-Chao Yu et al. used male mice when studying the therapeutic effect of IL-4 on periventricular leukomalacia to avoid the bias of gender in the experiment (50). In a detailed study of mice microglia, researchers found that a small cluster of extremely specific microglia is enriched in females, which is different from males, and sex only had little effect on microglia diversity (16). Further research should focus on the specific gender-related microglia and the mechanisms behind them.

Nowadays, treatments for cerebral palsy are mainly physiotherapy and rehabilitation. Neural stem cell transplantation seems to be a better choice for patients with CP recently. Researchers have applied stem cell therapy in neurodegenerative diseases or brain injury for its multi-differentiation potential and migration ability. Hoping that stem cells could reach the lesion, proliferate, differentiate, and finally reshape neurons binding (54–56). Besides, trophic factor supplement and immunomodulation are two critical keys for effective neural stem cell therapy (57). Since microglia are immunocompetent cells and important regulators of the cerebral microenvironment, our findings will shed light on the combination of immunomodulation and NSCs transplantation.

Our study also has certain limitations. The first limitation is that we only chose one marker protein for each type of microglia. There are other markers such as iNOS/CD16/CD86 for M1-type microglia and CD163/CD106 for M2-type microglia (27, 50). Perhaps different markers can be applied in a future study to improve experimental accuracy. The other limitation is that we only captured M2 polarization because of the long interval between LPS injections. In further research, by adding more experimental groups and shortening the interval between LPS injections, the critical time of the transition from M1 polarization to M2 polarization could be found.

In conclusion, the perinatal infection can induce WMI to CP based on our “multi-hit” animal model and microglia play a critical role in cerebral inflammation. The establishment of this model supports the “multi-hits” theory of CP formation. Our findings may shed some light on further mechanism research of microglia in CP and future treatment discovery.

## Data Availability Statement

The raw data supporting the conclusions of this article will be made available by the authors, without undue reservation.

## Ethics Statement

The animal study was reviewed and approved by Ethics Committee of Experimental Animals, Anhui Medical University.

## Author Contributions

LL and DW: study design. LL, BD, LF, YW, and ZC: study implementation. LF and LL: data analysis and figures preparation. LF, LL, DW, and LY manuscript drafting. All authors approved the final version of the manuscript.

## Conflict of Interest

The authors declare that the research was conducted in the absence of any commercial or financial relationships that could be construed as a potential conflict of interest.

## Publisher’s Note

All claims expressed in this article are solely those of the authors and do not necessarily represent those of their affiliated organizations, or those of the publisher, the editors and the reviewers. Any product that may be evaluated in this article, or claim that may be made by its manufacturer, is not guaranteed or endorsed by the publisher.

## References

[1] Sadowska M Sarecka-Hujar B Kopyta I. Cerebral palsy: current opinions on definition, epidemiology, risk factors, classification and treatment options. *Neuropsych Dis Treat.* (2020) 16:1505–18. 10.2147/ndt.S235165 32606703 PMC7297454

[2] Yang S Xia J Gao J Wang L. Increasing prevalence of cerebral palsy among children and adolescents in China 1988-2020: a systematic review and meta-analysis. *J Rehabil Med.* (2021) 53:jrm00195. 10.2340/16501977-2841 33961057 PMC8814846

[3] Finch-Edmondson M Morgan C Hunt RW Novak I. Emergent prophylactic, reparative and restorative brain interventions for infants born preterm with cerebral palsy. *Front Physiol.* (2019) 10:15. 10.3389/fphys.2019.00015 30745876 PMC6360173

[4] Oskoui M Coutinho F Dykeman J Jette N Pringsheim T. An update on the prevalence of cerebral palsy: a systematic review and meta-analysis. *Dev Med Child Neurol.* (2013) 55:509–19. 10.1111/dmcn.12080 23346889

[5] Wimalasundera N Stevenson VL. Cerebral palsy. *Pract Neurol.* (2016) 16:184–94. 10.1136/practneurol-2015-001184 26837375

[6] Novak I Morgan C Adde L Blackman J Boyd RN Brunstrom-Hernandez J Early, accurate diagnosis and early intervention in cerebral palsy: advances in diagnosis and treatment. *JAMA Pediatr.* (2017) 171:897–907. 10.1001/jamapediatrics.2017.1689 28715518 PMC9641643

[7] Shao R Sun D Hu Y Cui D. White matter injury in the neonatal hypoxic-ischemic brain and potential therapies targeting microglia. *J Neurosci Res.* (2021) 99:991–1008. 10.1002/jnr.24761 33416205

[8] Doyle LW Halliday HL Ehrenkranz RA Davis PG Sinclair JC. An update on the impact of postnatal systemic corticosteroids on mortality and cerebral palsy in preterm infants: effect modification by risk of bronchopulmonary dysplasia. *J Pediatr.* (2014) 165:1258–60. 10.1016/j.jpeds.2014.07.049 25217197

[9] Chung SH Biswas S Sohn J Jiang P Dehghan S Marzban H The P38alpha Mapk deletion in oligodendroglia does not attenuate myelination defects in a mouse model of periventricular leukomalacia. *Neuroscience.* (2018) 386:175–81. 10.1016/j.neuroscience.2018.06.037 29966722 PMC6076863

[10] Billiards SS Haynes RL Folkerth RD Borenstein NS Trachtenberg FL Rowitch DH Myelin abnormalities without oligodendrocyte loss in periventricular leukomalacia. *Brain Pathol.* (2008) 18:153–63. 10.1111/j.1750-3639.2007.00107.x 18177464 PMC2770329

[11] Tuzun F Gencpinar P Ozbal S Dilek M Ergur BU Duman N Neuroprotective effect of neotrofin in a neonatal rat model of periventricular leukomalacia. *Neurosci Lett.* (2012) 520:6–10. 10.1016/j.neulet.2012.04.076 22579826

[12] Guo K Yang Y Qiu J Kan Q Zhou XG Zhou XY. The expression profile of micrornas in wistar rats with lipopolysaccharide-induced periventricular leukomalacia. *J Mol Neurosci.* (2013) 51:941–9. 10.1007/s12031-013-9958-y 23354881

[13] Hu Y Chen G Wan H Zhang Z Zhi H Liu W A rat pup model of cerebral palsy induced by prenatal inflammation and hypoxia. *Neural Regen Res.* (2013) 8:817–24. 10.3969/j.issn.1673-5374.2013.09.006 25206729 PMC4146090

[14] Stigger F Lovatel G Marques M Bertoldi K Moyses F Elsner V Inflammatory response and oxidative stress in developing rat brain and its consequences on motor behavior following maternal administration of LPS and perinatal anoxia. *Int J Dev Neurosci.* (2013) 31:820–7. 10.1016/j.ijdevneu.2013.10.003 24140242

[15] Fragopoulou AF Qian Y Heijtz RD Forssberg H. Can neonatal systemic inflammation and hypoxia yield a cerebral palsy-like phenotype in periadolescent mice? *Mol Neurobiol.* (2019) 56:6883–900. 10.1007/s12035-019-1548-8 30941732 PMC6728419

[16] Hammond TR Dufort C Dissing-Olesen L Giera S Young A Wysoker A Single-cell Rna sequencing of microglia throughout the mouse lifespan and in the injured brain reveals complex cell-state changes. *Immunity.* (2019) 50:253–71e6. 10.1016/j.immuni.2018.11.004 30471926 PMC6655561

[17] Wlodarczyk A Cedile O Jensen KN Jasson A Mony JT Khorooshi R Pathologic and protective roles for microglial subsets and bone marrow- and blood-derived myeloid cells in central nervous system inflammation. *Front Immunol.* (2015) 6:463. 10.3389/fimmu.2015.00463 26441968 PMC4562247

[18] Franco R Fernandez-Suarez D. Alternatively activated microglia and macrophages in the central nervous system. *Prog Neurobiol.* (2015) 131:65–86. 10.1016/j.pneurobio.2015.05.003 26067058

[19] Xiong XY Liu L Yang QW. Functions and mechanisms of microglia/macrophages in neuroinflammation and neurogenesis after stroke. *Prog Neurobiol.* (2016) 142:23–44. 10.1016/j.pneurobio.2016.05.001 27166859

[20] Gonzalez-Reyes RE Nava-Mesa MO Vargas-Sanchez K Ariza-Salamanca D Mora-Munoz L. Involvement of astrocytes in Alzheimer’s disease from a neuroinflammatory and oxidative stress perspective. *Front Mol Neurosci.* (2017) 10:427. 10.3389/fnmol.2017.00427 29311817 PMC5742194

[21] Pol SU Polanco JJ Seidman RA O’Bara MA Shayya HJ Dietz KC Network-based genomic analysis of human oligodendrocyte progenitor differentiation. *Stem Cell Rep.* (2017) 9:710–23. 10.1016/j.stemcr.2017.07.007 28793249 PMC5550273

[22] Sherwin C Fern R. Acute lipopolysaccharide-mediated injury in neonatal white matter glia: role of Tnf-Alpha, Il-1beta, and calcium. *J Immunol.* (2005) 175:155–61. 10.4049/jimmunol.175.1.155 15972642

[23] Pang Y Campbell L Zheng B Fan L Cai Z Rhodes P. Lipopolysaccharide-activated microglia induce death of oligodendrocyte progenitor cells and impede their development. *Neurosci.* (2010) 166:464–75. 10.1016/j.neuroscience.2009.12.040 20035837

[24] Graf AE Haines KM Pierson CR Bolon BN Houston RH Velten M Perinatal inflammation results in decreased oligodendrocyte numbers in adulthood. *Life Sci.* (2014) 94:164–71. 10.1016/j.lfs.2013.11.015 24291255 PMC3923532

[25] Zhang Y Argaw AT Gurfein BT Zameer A Snyder BJ Ge C Notch1 signaling plays a role in regulating precursor differentiation during Cns remyelination. *Proc Natl Acad Sci USA.* (2009) 106:19162–7. 10.1073/pnas.0902834106 19855010 PMC2776461

[26] Yuan TM Yu HM. Notch signaling: key role in intrauterine infection/inflammation, embryonic development, and white matter damage? *J Neurosci Res.* (2010) 88:461–8. 10.1002/jnr.22229 19768798

[27] Deng XL Feng L Wang ZX Zhao YE Zhan Q Wu XM The Runx1/Notch1 signaling pathway participates in M1/M2 microglia polarization in a mouse model of temporal lobe epilepsy and in Bv-2 cells. *Neurochem Res.* (2020) 45:2204–16. 10.1007/s11064-020-03082-3 32632543

[28] Schaar KL Brenneman MM Savitz SI. Functional assessments in the rodent stroke model. *Exp Translat Stroke Med.* (2010) 2:13. 10.1186/2040-7378-2-13 20642841 PMC2915950

[29] Gómez C Carrasco C Redolat R. Adolescent and adult mice display differential sensitivity to the effects of bupropion on the acquisition of a water maze task. *Pharmacol Rep PR.* (2017) 69:162–7. 10.1016/j.pharep.2016.10.008 27923160

[30] Ito D Imai Y Ohsawa K Nakajima K Fukuuchi Y Kohsaka S. Microglia-specific localisation of a novel calcium binding protein, Iba1. *Brain Res Mol Brain Res.* (1998) 57:1–9. 10.1016/s0169-328x(98)00040-0 9630473

[31] Morgan C Novak I Dale RC Badawi N. Optimising motor learning in infants at high risk of cerebral palsy: a pilot study. *BMC Pediatr.* (2015) 15:30. 10.1186/s12887-015-0347-2 25880227 PMC4389951

[32] Koob M Viola A Le Fur Y Viout P Ratiney H Confort-Gouny S Creatine, glutamine plus glutamate, and macromolecules are decreased in the central white matter of premature neonates around term. *PLoS One.* (2016) 11:e0160990. 10.1371/journal.pone.0160990 27547969 PMC4993494

[33] Yang L Sameshima H Ikeda T Ikenoue T. Lipopolysaccharide administration enhances hypoxic-ischemic brain damage in newborn rats. *J Obstetr Gynaecol Res.* (2004) 30:142–7. 10.1111/j.1447-0756.2003.00174.x 15009619

[34] Eklind S Mallard C Arvidsson P Hagberg H. Lipopolysaccharide induces both a primary and a secondary phase of sensitization in the developing rat brain. *Pediatr Res.* (2005) 58:112–6. 10.1203/01.Pdr.0000163513.03619.8d 15879289

[35] Leviton A Fichorova RN O’Shea TM Kuban K Paneth N Dammann O Two-Hit model of brain damage in the very preterm newborn: small for gestational age and postnatal systemic inflammation. *Pediatr Res.* (2013) 73:362–70. 10.1038/pr.2012.188 23364171 PMC3642985

[36] Korzeniewski SJ Romero R Cortez J Pappas A Schwartz AG Kim CJ A “Multi-Hit” model of neonatal white matter injury: cumulative contributions of chronic placental inflammation, acute fetal inflammation and postnatal inflammatory events. *J Perinatal Med.* (2014) 42:731–43. 10.1515/jpm-2014-0250 25205706 PMC5987202

[37] Yanni D Korzeniewski SJ Allred EN Fichorova RN O’Shea TM Kuban K Both antenatal and postnatal inflammation contribute information about the risk of brain damage in extremely preterm newborns. *Pediatr Res.* (2017) 82:691–6. 10.1038/pr.2017.128 28549057 PMC5599336

[38] Barnett ML Tusor N Ball G Chew A Falconer S Aljabar P Exploring the multiple-hit hypothesis of preterm white matter damage using diffusion Mri. *Neuroimage Clin.* (2018) 17:596–606. 10.1016/j.nicl.2017.11.017 29234596 PMC5716951

[39] Mor O Stavsky M Yitshak-Sade M Mastrolia SA Beer-Weisel R Rafaeli-Yehudai T Early onset preeclampsia and cerebral palsy: a double hit model? *Am J Obstetr Gynecol.* (2016) 214:.e1–9. 10.1016/j.ajog.2015.08.020 26283455

[40] Mallard C Hagberg H. Inflammation-induced preconditioning in the immature brain. *Semin Fetal Neonatal Med.* (2007) 12:280–6. 10.1016/j.siny.2007.01.014 17327146

[41] Kumral A Tuzun F Ozbal S Ugur Ergur B Yılmaz O Duman N Lipopolysaccharide-preconditioning protects against endotoxin-induced white matter injury in the neonatal rat brain. *Brain Res.* (2012) 1489:81–9. 10.1016/j.brainres.2012.10.015 23063716

[42] Salter MW Stevens B. Microglia emerge as central players in brain disease. *Nat Med.* (2017) 23:1018–27. 10.1038/nm.4397 28886007

[43] He LF Chen HJ Qian LH Chen GY Buzby JS. Curcumin protects pre-oligodendrocytes from activated microglia in Vitro and in Vivo. *Brain Res.* (2010) 1339:60–9. 10.1016/j.brainres.2010.04.014 20403340

[44] Rochefort N Quenech’du N Watroba L Mallat M Giaume C Milleret C. Microglia and astrocytes may participate in the shaping of visual callosal projections during postnatal development. *J Physiol Paris.* (2002) 96:183–92. 10.1016/s0928-4257(02)00005-0 12445895

[45] Vasung L Huang H Jovanov-Milosevic N Pletikos M Mori S Kostovic I. Development of axonal pathways in the human fetal fronto-limbic brain: histochemical characterization and diffusion tensor imaging. *J Anat.* (2010) 217:400–17. 10.1111/j.1469-7580.2010.01260.x 20609031 PMC2992416

[46] Verney C Pogledic I Biran V Adle-Biassette H Fallet-Bianco C Gressens P. Microglial reaction in axonal crossroads is a hallmark of noncystic periventricular white matter injury in very preterm infants. *J Neuropathol Exp Neurol.* (2012) 71:251–64. 10.1097/NEN.0b013e3182496429 22318128

[47] Verney C Monier A Fallet-Bianco C Gressens P. Early microglial colonization of the human forebrain and possible involvement in periventricular white-matter injury of preterm infants. *J Anat.* (2010) 217:436–48. 10.1111/j.1469-7580.2010.01245.x 20557401 PMC2992419

[48] Biber K Owens T Boddeke E. What is microglia neurotoxicity (Not)? *Glia.* (2014) 62:841–54. 10.1002/glia.22654 24590682

[49] Norden DM Trojanowski PJ Villanueva E Navarro E Godbout JP. Sequential activation of microglia and astrocyte cytokine expression precedes increased Iba-1 or Gfap immunoreactivity following systemic immune challenge. *Glia.* (2016) 64:300–16. 10.1002/glia.22930 26470014 PMC4707977

[50] Yu LC Miao JK Li WB Chen N Chen QX. Intranasal Il-4 administration alleviates functional deficits of periventricular leukomalacia in neonatal mice. *Front Neurol.* (2020) 11:930. 10.3389/fneur.2020.00930 32982939 PMC7492203

[51] Ment LR Vohr B Allan W Katz KH Schneider KC Westerveld M Change in cognitive function over time in very low-birth-weight infants. *JAMA.* (2003) 289:705–11. 10.1001/jama.289.6.705 12585948

[52] Al Mamun A Yu H Romana S Liu F. Inflammatory responses are sex specific in chronic hypoxic-ischemic encephalopathy. *Cell Transplant.* (2018) 27:1328–39. 10.1177/0963689718766362 29692197 PMC6168990

[53] Carlezon WA Jr. Kim W Missig G Finger BC Landino SM Alexander AJ Maternal and early postnatal immune activation produce sex-specific effects on autism-like behaviors and neuroimmune function in mice. *Sci Rep.* (2019) 9:16928. 10.1038/s41598-019-53294-z 31729416 PMC6858355

[54] Kiasatdolatabadi A Lotfibakhshaiesh N Yazdankhah M Ebrahimi-Barough S Jafarabadi M Ai A The role of stem cells in the treatment of cerebral palsy: a review. *Mol Neurobiol.* (2017) 54:4963–72. 10.1007/s12035-016-0030-0 27520277

[55] Jantzie LL Scafidi J Robinson S. Stem cells and cell-based therapies for cerebral palsy: a call for rigor. *Pediatr Res.* (2018) 83:345–55. 10.1038/pr.2017.233 28922350 PMC12917736

[56] Jiao Y Li XY Liu J. A new approach to cerebral palsy treatment: discussion of the effective components of umbilical cord blood and its mechanisms of action. *Cell Transplant.* (2019) 28:497–509. 10.1177/0963689718809658 30384766 PMC7103597

[57] Bennet L Tan S Van den Heuij L Derrick M Groenendaal F van Bel F Cell therapy for neonatal hypoxia-ischemia and cerebral palsy. *Ann Neurol.* (2012) 71:589–600. 10.1002/ana.22670 22522476

